# Effect of mastectomy on gut microbiota and its metabolites in patients with breast cancer

**DOI:** 10.3389/fmicb.2024.1269558

**Published:** 2024-05-27

**Authors:** Pingming Fan, Linwei Ding, Guankui Du, Changyuan Wei

**Affiliations:** ^1^Department of Breast Surgery, Guangxi Medical University Cancer Hospital, Nanning, China; ^2^Department of Breast Surgery, The First Affiliated Hospital of Hainan Medical University, Haikou, China; ^3^Department of Biochemistry and Molecular Biology, Hainan Medical University, Haikou, China

**Keywords:** breast cancer, surgery, gut microbiota, metabolites, 16s rRNA

## Abstract

**Background:**

The relationship between gut microbiota and breast cancer has been extensively studied; however, changes in gut microbiota after breast cancer surgery are still largely unknown.

**Materials and methods:**

A total of 20 patients with breast cancer underwent routine open surgery at the First Affiliated Hospital of Hainan Medical College from 1 June 2022 to 1 December 2022. Stool samples were collected from the patients undergoing mastectomy for breast cancer preoperatively, 3 days later, and 7 days later postoperatively. The stool samples were subjected to 16s rRNA sequencing.

**Results:**

Surgery did not affect the α-diversity of gut microbiota. The β-diversity and composition of gut microorganisms were significantly affected by surgery in breast cancer patients. Both linear discriminant analysis effect size (LEfSe) analysis and between-group differences analysis showed that surgery led to a decrease in the abundance of *Firmicutes* and *Lachnospiraceae* and an increase in the abundance of *Proteobacteria* and *Enterobacteriaceae*. Moreover, 127 differential metabolites were screened and classified into 5 categories based on their changing trends. The Kyoto Encyclopedia of Genes and Genomes (KEGG) enrichment analysis showed significant changes in the phenylalanine metabolic pathway and exogenous substance metabolic pathway. Eight characterized metabolites were screened using ROC analysis.

**Conclusion:**

Our study found that breast cancer surgery significantly altered gut microbiota composition and metabolites, with a decrease in beneficial bacteria and an increase in potentially harmful bacteria. This underscores the importance of enhanced postoperative management to optimize gut microbiota.

## Introduction

Breast cancer is a common malignant tumor, with an increasing incidence year by year (Azamjah et al., 2019). According to the latest data released by the World Health Organization, there are approximately 2.3 million new cases of breast cancer globally each year, with women accounting for 99.5%. An estimated 685,000 women died from breast cancer in 2020 (Arnold et al., 2022). The incidence rate and mortality rate of breast cancer in some developed countries and regions are relatively high, which may be related to lifestyle and eating habits (Yap et al., 2019). Meanwhile, many challenges exist in developing countries and regions, including insufficient medical resources and outdated technology, which further exacerbate the impact of breast cancer (Ghoncheh et al., 2016).

Mastectomy is a surgical procedure that primarily aims to preserve the appearance and function of the breast while removing the tumor and its surrounding tissue (ElSherif et al., 2023). Breast preservation surgery is indicated for patients with early-stage breast cancer and typically requires a combination of radiation and chemotherapy to reduce the risk of recurrence. Although mastectomy is an effective treatment for breast cancer, patients may experience postoperative depression, which can stem from several reasons, including changes in body image, fear of the disease, pain, and discomfort (Wu et al., 2023). Additionally, during radical breast cancer surgery, if the aseptic operation is not strictly followed or if the patient’s own body resistance is relatively poor, postoperative sequelae such as wound infection and prolonged wound healing may occur (Jorgensen et al., 2023). If the patient’s condition is relatively serious and the pectoralis major muscle is injured during the operation, muscle abnormalities may occur, and vitamin B1 and vitamin B12 supplementation are necessary (Punchai et al., 2017). Therefore, post-mastectomy patients are required to pay special attention to multiple aspects, including wound care, psychological adjustment, and dietary adjustment.

The gut microbiota plays an important role in maintaining host health (Yang et al., 2023). The prevalence of bacterial infection in patients with breast cancer is typically characterized by reduced microbial diversity and changes in microbial composition (Alvarez-Mercado et al., 2023; Caleca et al., 2023). The gut microbiota can produce a multitude of metabolites that interact with the host, including vitamins B12, 5-hydroxytryptamine, and phenylalanine (Kiecka et al., 2023). A clinical study revealed a significant change in the total number of fecal bacteria and the absolute counts of *Proteobacteria*, *Firmicutes*, and *Actinobacteria* in breast cancer patients (Toumazi et al., 2021). It has been shown that gut microbes are involved in estrogen metabolism, determine estrogen levels in the circulatory system, and may also be responsible for the development of breast cancer (Feng et al., 2021; Marino et al., 2022).

However, there is a lack of knowledge regarding the impact of mastectomy on the gut microbiota and its metabolites in breast cancer patients. The aim of this paper is to investigate the alterations in the diversity and composition of the gut microbiota of breast cancer patients after surgery, as well as to analyze the changes in microbial metabolites using the metabolome technique.

## Materials and methods

### Patients

A total of 20 patients with breast cancer underwent open surgery at the First Affiliated Hospital of Hainan Medical College between 1 June 2022 and 1 December 2022. The patients ranged in age from 38 to 76 years, with a mean age of 52.01, and their BMI ranged from 18.2 to 33.3, with a mean value of 24.16 (Table 1). Prior to sampling, none of the patients had received antibiotic treatment for at least a month.

**Table 1:** Basic information of patients.

Age	51.01 ± 12.07
BMI	23.66 ± 3.81
Height	157.36 ± 4.29
Weight	59.6 ± 11.42
*Pathological type*	
Non-specific invasive cancer	10
Invasive lobular carcinoma	4
Other invasive cancers	6
Pathological grade	
Grade III	4
Grade II	14
Grade I	2
*Lymph node metastasis*	
Transferred	10
Not transferred	10

Fecal samples were collected from patients undergoing mastectomy for breast cancer preoperatively (KF0 group), 3 days later (KF3 group), and 7 days later (KF7 group) postoperatively. A total of 60 samples were collected, each containing 5 g. After collection, the stool samples were quickly frozen in liquid nitrogen and transferred to a refrigerator at −80°C for storage.

### DNA extraction and 16s rRNA sequencing

The DNA of the sample was extracted using the CTAB method. The concentration of the DNA was measured using a NanoDrop 2000. A suitable amount of the sample was taken into a centrifuge tube and diluted with sterile water to a concentration of 1 ng/ul. The primers for the 16S rDNA V4 region were as follows: 515F: GTGCCAGCMGCCGCGGTAA; 806R: GGACTACNNGGGGTATCTAAT. A library was constructed using the TruSeqR DNA PCR Free Sample Preparation Kit. The library was quantified using a Life Invitrogen Qubit 3.0 and library assay. The sequencing was performed on a HiSeq2500 platform after passing the assay.

### Analysis of 16s rRNA sequencing data

All analyses of the data were performed on the Majorbio platform.^1^ Sequence splicing between bipartite pairs was conducted using the Flash software. The abundance tables for each taxonomy were generated using QIIME software and calculated using beta diversity distances. The OTU statistics were performed using Usearch. The rRNA database comparison annotation was performed by Greengenes. Intergroup difference analysis was performed using the Wilcoxon rank-sum test. Bacterial taxa with significant differences in abundance from phylum to genus level between groups were identified using the linear discriminant analysis effect size (LEfSe) (LDA > 2, *p* < 0.05).

### Statistical methods

Statistical analyses were conducted using SPSS 21.0 and Excel. Data were expressed as means ± standard deviations and compared using an independent sample t-test. A one-way ANOVA was used to compare multiple groups, with *p*-values set at <0.05. The Chi-square test was used for counting information, with *p*-values set at <0.05.

## Results

### Effect of mastectomy on the α-diversity of gut microbiota in breast cancer patients

**Figure 1:**
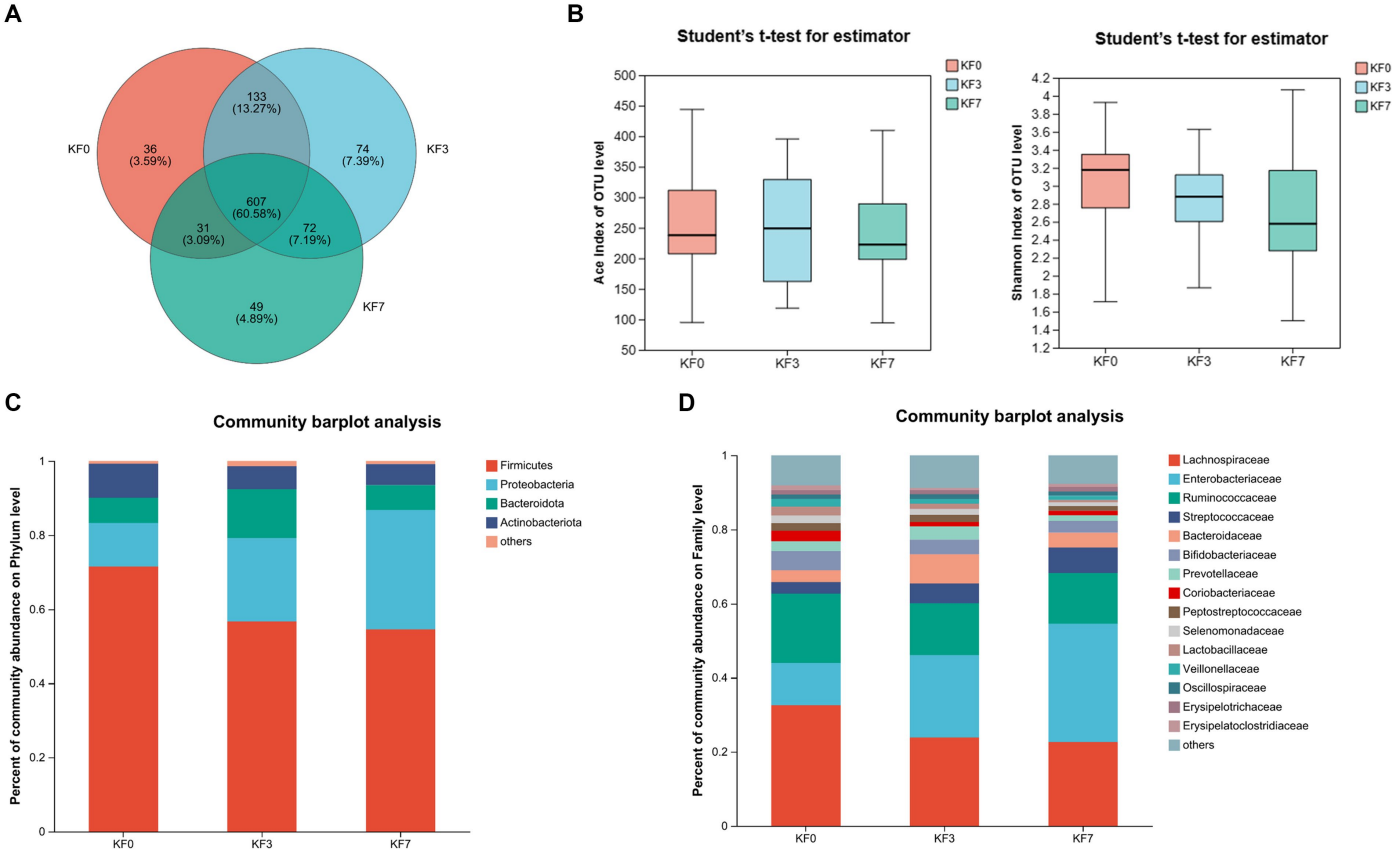
Changes in the α-diversity and composition of the gut microbiota of patients after open surgery in breast cancer patients. **(A)** Venn diagram of gut microbiota composition analysis. **(B)** Alpha diversity analysis, including the ACE index and Shannon index. Gut microbiota composition at **(C)** phylum level and **(D)** family level.

To study the effect of mastectomy on the gut microbiota of breast cancer patients, fecal samples from patients before and after surgery were collected and analyzed using 16s rRNA sequencing. The operational taxonomic unit (OTU) analysis showed that there were 807, 886, and 759 OTUs in the KF0, KF3, and KF7 groups, respectively (Figure 1A). The α-diversity analysis of the gut microbiota of patients 3 and 7 days after surgery showed that the ACE index and Shannon index were not significantly different among groups (Figure 1B).

### Effect of mastectomy on the composition of gut microbiota in breast cancer patients

The composition of the intestinal microbiota community of the patients was further analyzed. The dominant bacteria at the phylum level were *Firmicutes*, *Proteobacteria*, *Bacteroides*, and *Actinobacteria* (Figure 1C). The abundance of *Firmicutes* and *Proteobacteria* accounted for 83.31% of the total phyla. Moreover, *Lachnospiraceae*, *Enterobacteriaceae*, and *Ruminococcaceae* were the dominant families, with the abundance of these three families accounting for more than 60% of the total (Figure 1D).

To analyze the effect of surgery on the β-diversity of the gut microbiota of breast cancer patients, the Partial Least-Squares Discriminant Analysis (PLS-DA) was performed. The KF0, KF3, and KF7 groups were partially overlapping and could be distinguished from each other (Supplementary Figure S1).

**Figure 2:**
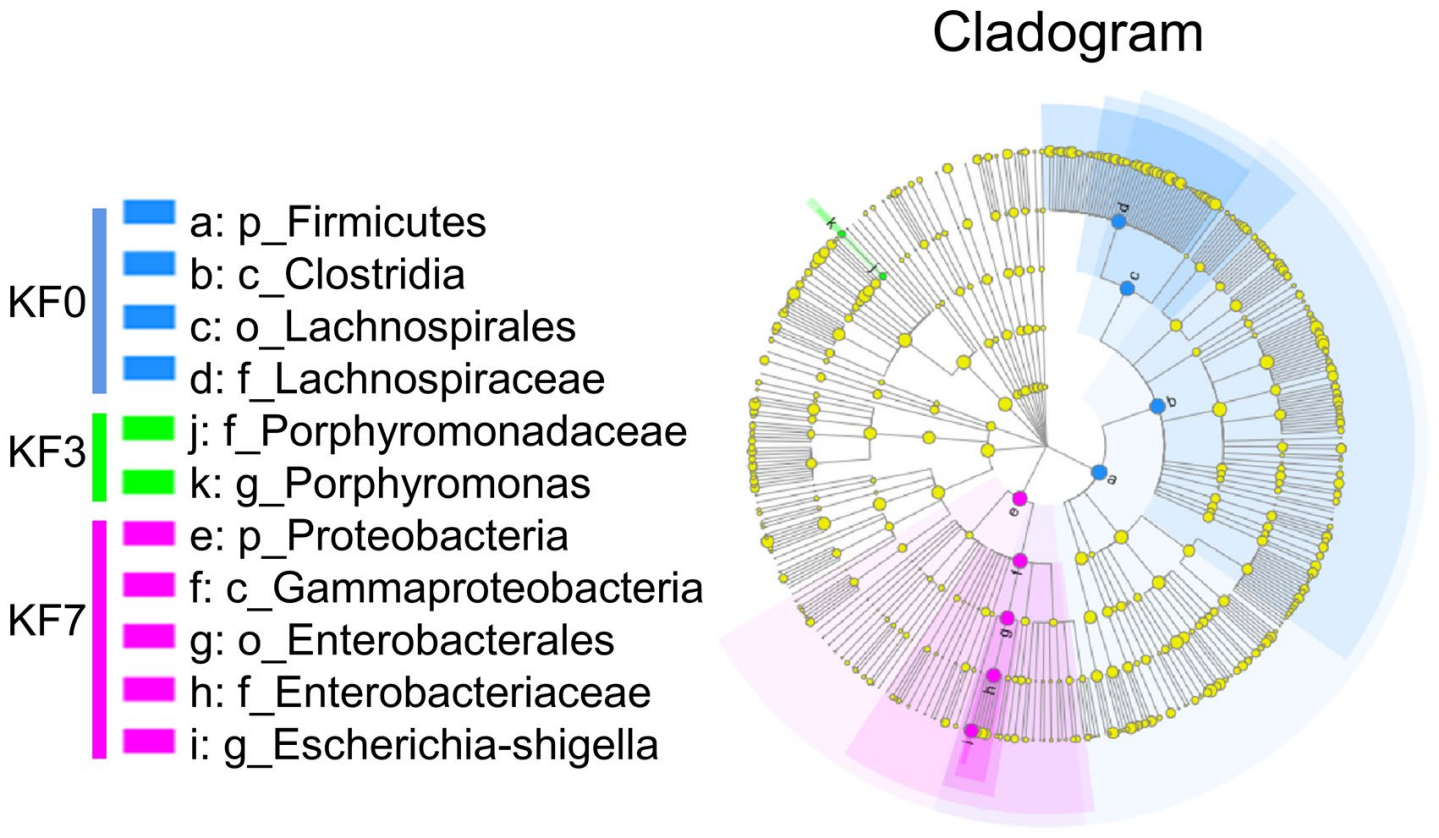
β-Diversity analysis after open surgery in breast cancer patients.

Screening of characteristic bacteria using the LEfSe analysis led to the identification of two groups of significantly different bacteria in abundance, including *p_Firmicutes-c_Clostridia-o_Lachnospirale-f_Lachnospiraceae* and *p_Proteobacteria-c_Gammaproteobacteria-o_Enterobacterales-f_Enterobacteriaceae* (Figure 2).

**Figure 3:**
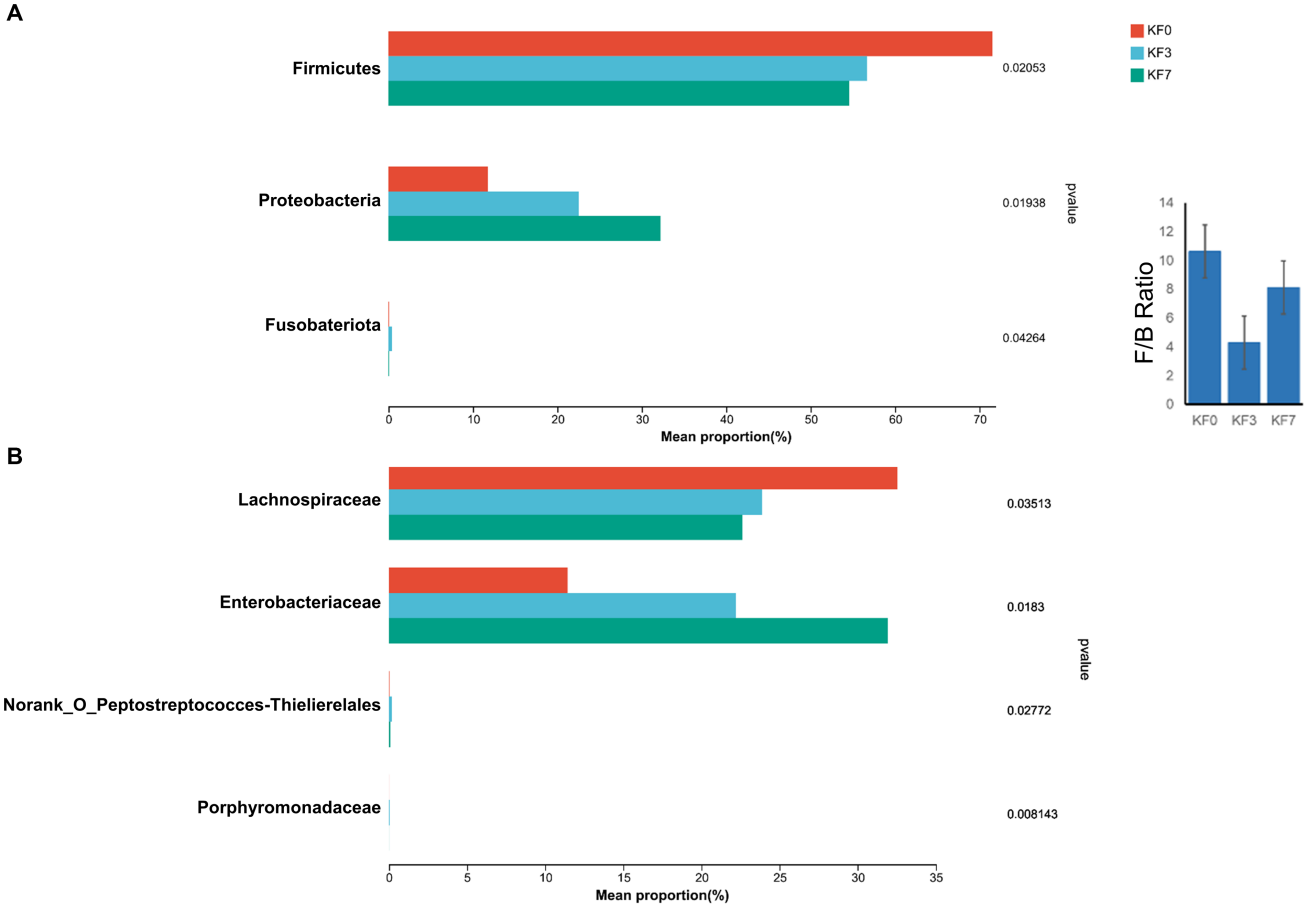
Differential bacterial analysis after open surgery in breast cancer patients. **(A)** Phylum level and **(B)** Family level after open surgery.

To identify bacteria with significant differences, the Kruskal–Wallis H-test analysis was performed. Significant differences were found in three bacterial phyla. *Firmicutes* were significantly decreased, while *Proteobacteria* and *Fusobacteriotes* were significantly increased (Figure 3A). Additionally, the ratio of *Firmicutes* to *Bacteroides* was significantly lower in the KF3 and KF7 groups than the KF0 group. At the family level, the abundance of *Lachnospiraceae* was significantly decreased, while that of *Enterobacteriaceae* and *Norank_O_Peptostreptococces-Thielierelales* was significantly increased (Figure 3B).

### Effect of mastectomy on the metabolism of gut microbiota in patients with breast cancer

As shown in Supplementary Figure S2, significant differences in metabolite concentrations between groups were demonstrated using volcano plots. In the positive ion mode, 102 different metabolites were identified between KF0 and KF3, while 91 different metabolites were observed between KF0 and KF7. In the negative ion mode, 25 different metabolites were observed in KF3, and 54 different metabolites were found in KF7, compared to KF0.

**Figure 4:**
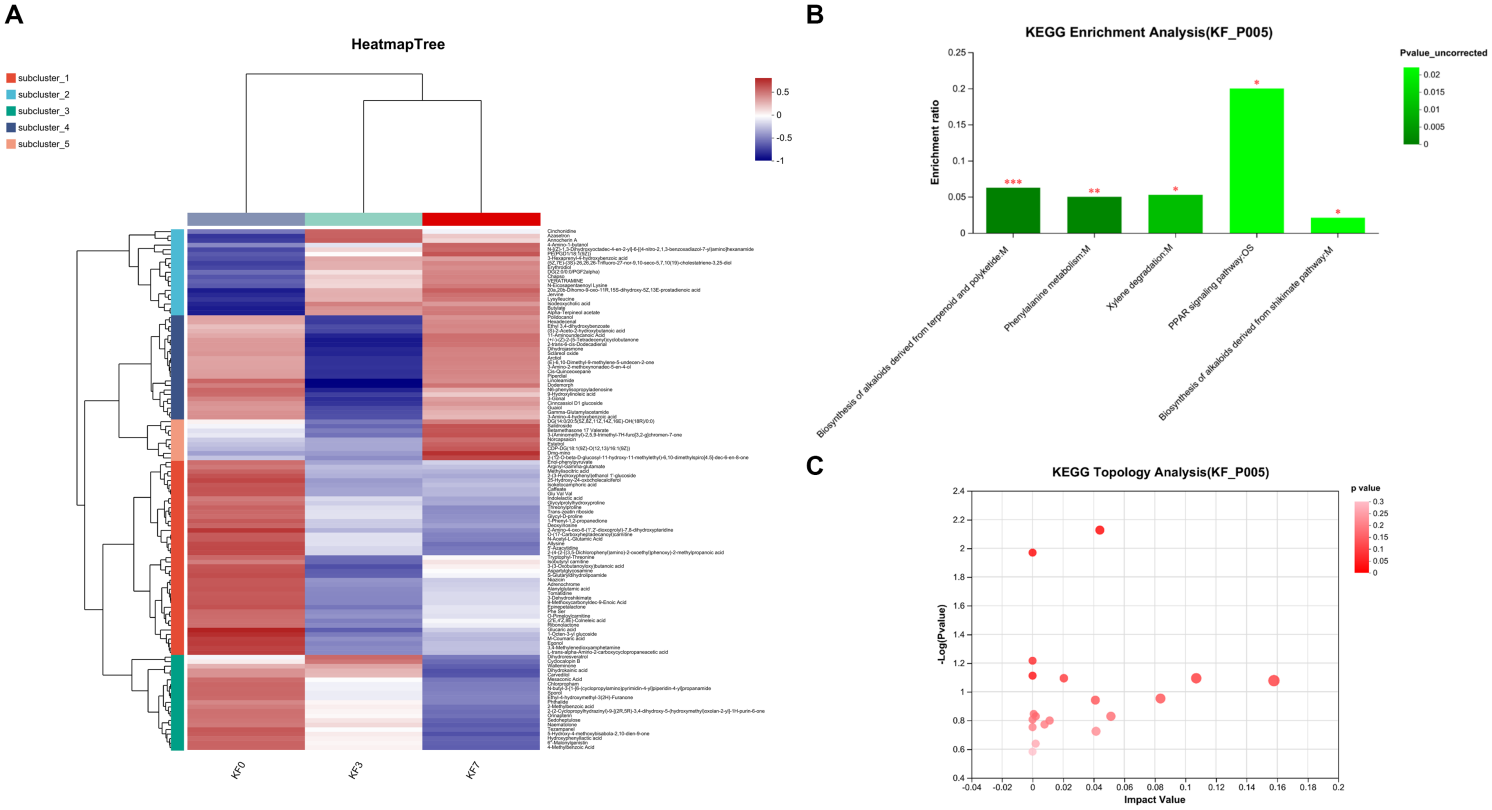
Changes in the metabolites of gut microbiota after open surgery in breast cancer patients. **(A)** Differential metabolite clustering analysis. **(B)** KEGG functional pathway enrichment analysis and **(C)** KEGG topology analysis.

As shown in Figure 4A, the clustering analysis of differential metabolites can be divided into five clusters. Cluster 1 consisted of 43 metabolites, including phenylpyruvic acid enol, arginine γ-glutamic acid, methylisocitric acid, 2-(3-hydroxyphenyl)ethanol 1′-glucose, and 25-hydroxy-24-oxocholecalciferol, whose abundance decreased after surgery. Cluster 2 consisted of 19 metabolites, including cinchonidine, lysine leucine, isodeoxycholic acid, butyl ester, and alpha-pinoresinol acetate, whose abundance increased after surgery. Cluster 3 consisted of 21 metabolites, including phthalates, dihydroresveratrol, 2-methylbenzoic acid, and 4-methylbenzoic acid, whose abundance decreased with postoperative time. Cluster 4 consisted of 23 metabolites, including 9-hydroxylinoleic acid, γ-glutamyl acetamide, and linoleic acid amide, whose abundance decreased significantly at 3 days postoperatively and returned to preoperative levels at 7 days postoperatively. Cluster 5 consisted of 9 metabolites, including rhodiola rosea glycosides and desmethyl capsaicin, which significantly increased in abundance 7 days postoperatively.

The analysis of the KEGG functional pathway indicated that five significant signaling pathways were enriched, namely the biosynthesis of terpene and polyketide alkaloids, phenylalanine metabolism, xylene degradation, the PPAR signaling pathway, and the mangiferic acid alkaloid biosynthesis pathway (Figure 4B). The topology analysis also showed significant changes in the phenylalanine metabolism and xylene degradation pathways (Figure 4C).

**Figure 5:**
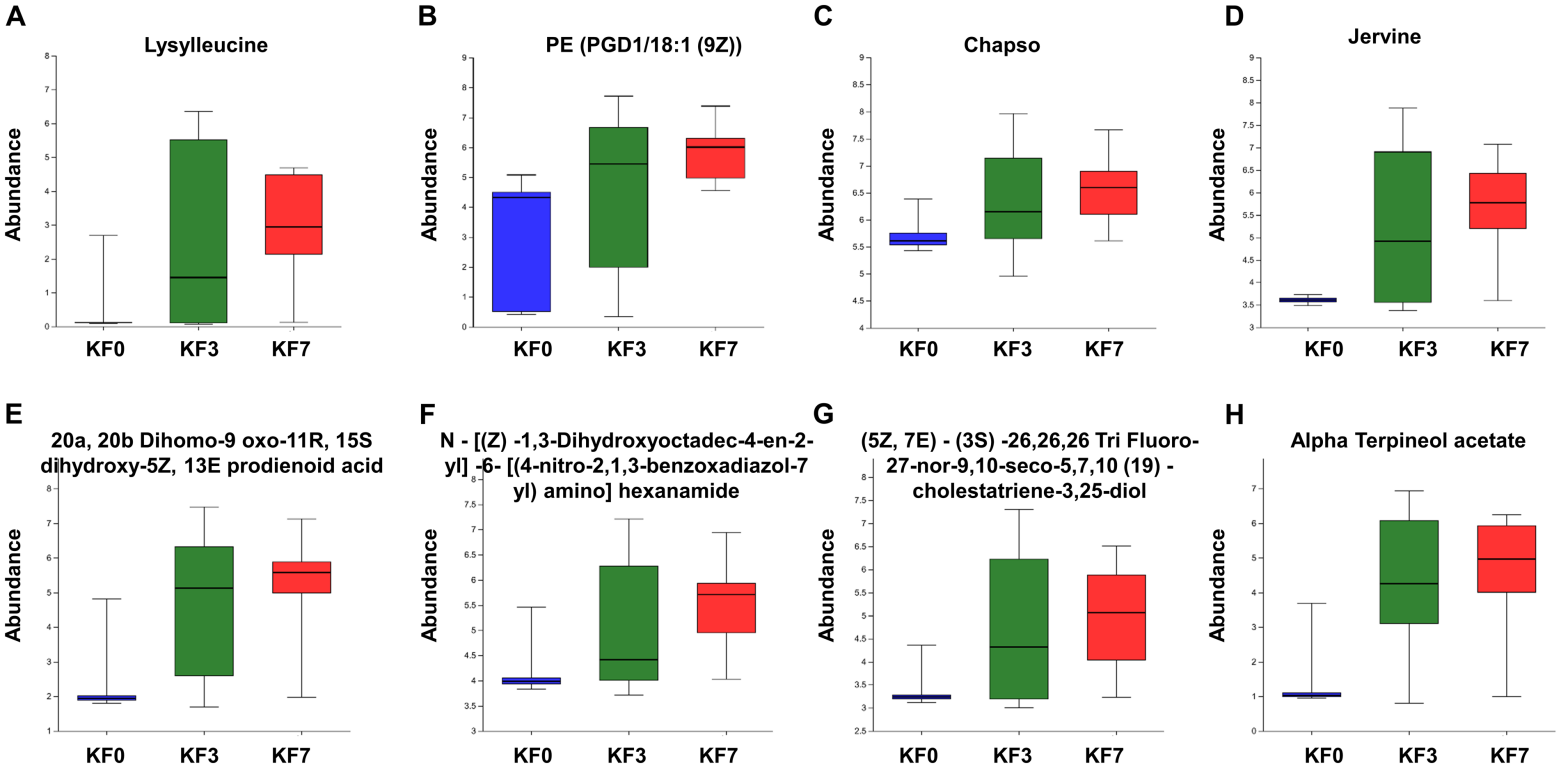
Abundance of characteristic metabolites. **(A)** Lysylleucine, **(B)** PE(PGD1/18:1(9Z)), **(C)** 20a,20b-dihomo-9-oxo-11R,15S-dihydroxy-5Z,13E-prostadienoic acid, **(D)** N-[(Z)-1,3-dihydroxyoctadec-4-en-2-yl]-6-[(4-nitro-2,1,3-benzoxadiazol-7-yl)amino]hexanamide, **(E)** Jervine, **(F)** (5Z,7E)-(3S)-26,26,26-trifluoro-27-nor-9,10-seco-5,7,10(19)-cholestatriene-3,25-diol, **(G)** Chapso, and **(H)** Alpha-terpineol acetate.

The ROC analysis identified eight important differential metabolites (Supplementary Figure S3), namely Lysylleucine, PE(PGD1/18:1 (9Z)), 20a,20b-dihomo-9-oxo-11R,15S-dihydroxy-5Z,13E-prodienoid acid, N-[(Z)-1,3-dihydroxyoctadec-4-en-2-yl]-6-[(4-nitro-2,1,3-benzoxadiazol-7-yl)amino]hexanamide, Jervine, (5Z,7E)-(3S)-26,26,26-tri-fluoro-27-nor-9,10-seco-5,7,10 (19)-cholestatriene-3,25-diol, Chapso, and Alpha-terpineol acetate. The abundance of these eight differential metabolites gradually increased at 3 and 7 days after surgery, compared to before surgery (Figure 5).

## Discussion

Several studies have demonstrated a correlation between the gut microbiota and breast cancer. These studies have demonstrated that the microbiota can enhance the metabolism of estrogen and induce inflammation (Bodai and Nakata, 2020; Arnone and Cook, 2022). The aim of this study is to analyze the changes in the gut microbiota and its metabolite abundance in patients with breast cancer following mastectomy.

A limited number of studies have analyzed changes in the microbiota after surgical treatment for oncologic diseases (Tong et al., 2020; Chen et al., 2022; Hajjar et al., 2023). Research has shown that the microbial community after surgery is similar to that of healthy individuals, which can promote the recovery of tumor patients (Wolfe and Markey, 2022). A follow-up study found that changes in the gut microbiota can be considered potential biomarkers for tumor recurrence and can be used to stratify the risk of recurrence (Peters et al., 2019; Huo et al., 2022). It has also been shown that surgical procedures lead to higher rates of infection and are an important factor affecting the gut microbiota (Mu et al., 2019). Clinical trials have reported an increase in potential pathogenic bacteria after surgery (Mu et al., 2019). An animal study showed that cecum suturing induced postoperative intestinal obstruction, postoperative bacterial dysbiosis, and changes in β-diversity in guinea pigs (Wang et al., 2019). In the present study, α-diversity had no significant change, while the PLS-DA analysis showed that the KF3 and KF7 groups were significantly different from the KF0 group. Therefore, this study highlights that mastectomy for breast cancer can affect the diversity of the gut microbiota.

*Firmicutes* and *Bacteroides* are the two primary phyla of the gut microbiota in healthy adults, representing approximately 90% of the known gut microbiota (Sun et al., 2022). *Firmicutes* is crucial for maintaining human homeostasis, and dietary fiber is a significant factor influencing the abundance of *Firmicutes* in the intestine (Sun et al., 2022). *Firmicutes* can directly or indirectly utilize dietary fiber and produce metabolites such as acetic acid, butyric acid, and lactic acid during proliferation (James et al., 2022; Sun et al., 2022). *Firmicutes* can affect the host’s inflammatory response, intestinal permeability, glucose metabolism, fatty acid metabolism, and tryptophan metabolism (Sun et al., 2022). The *Firmicutes*–*Bacteroides* ratio was significantly lower in patients with breast cancer than in healthy controls (An et al., 2023). *Lachnospiraceae* can hydrolyze starch and other sugars, producing butyrate and other SCFAs (Zhang et al., 2019; Li et al., 2023). The reduced abundance of *Lachnospiraceae* and the resulting low butyrate production may be important in triggering the recurrence of colitis (Sasaki et al., 2019). *Lachnospiraceae* is positively correlated with the serum level of docosahexaenoic acid, which may reduce the risk of coronary heart disease death and breast cancer development (Toya et al., 2020; Memili et al., 2023). In the present study, the abundance of *Firmicutes* was significantly reduced in the KF3 and KF7 groups, along with a significant decrease in the *Firmicutes*–*Bacteroides* ratio. Meanwhile, the abundance of *Lachnospiraceae* decreased after surgery. Therefore, postoperative dietary modifications, such as dietary fiber intake, can be an important means to regulate the gut microbiota and promote the postoperative recovery of patients.

*Proteobacteria* is a widespread, harmful phylum that is associated with the development of various diseases (Rizzatti et al., 2017). *Proteobacteria* is more prevalent in patients with non-alcoholic fatty liver disease (NAFLD), and it is an important causative agent of liver injury (Suppli et al., 2021). *Proteobacteria* are positively associated with intestinal inflammation (Lin et al., 2023). Levels of *Proteobacteria* are elevated in mice with colitis (Liu et al., 2023). Enterobacteriaceae is a family of Gram-negative bacteria that contains multiple species that are resistant to carbapenems, which can lead to an increase in mortality rates for various diseases, such as septic shock, bacteremia, endophthalmitis, and liver abscesses (Santos-Marques et al., 2022). *Enterobacteriaceae* was significantly increased in the type 2 diabetes group (Penckofer et al., 2020). In the present study, *Proteobacteria* and *Enterobacteriaceae* were significantly increased in the KF3 and KF7 groups. Therefore, intervention to suppress intestinal pathogenic bacteria may be a challenge for breast cancer surgery as well.

It has been observed that some patients with breast cancer exhibit emotional changes after undergoing surgery, including depressive symptoms (Wu et al., 2023). Breast cancer surgery is a major physical trauma for the patient, and coupled with the fear of cancer and uncertainty about their future, they are prone to mood fluctuations. Gut microbes are involved in the synthesis and metabolism of neurotransmitters such as phenylalanine, serotonin, and dopamine (Xu et al., 2022). Phenylalanine is a precursor of the catecholamines epinephrine, norepinephrine, and dopamine. A recent study showed that phenylalanine may be involved in regulating the mental health of patients (Akimitsu et al., 2013). It has been shown that phenylalanine is significantly reduced in depressed patients (Sharman et al., 2012). In this study, the metabolomic analysis revealed that the phenylalanine metabolic pathway was significantly inhibited. Therefore, rational modification of the postoperative diet, such as increasing amino acid levels such as phenylalanine, might be a viable option to regulate the postoperative mental status of patients with breast cancer.

## Conclusion

In summary, our results indicated that the diversity and composition of a patient’s gut microbiota were significantly affected 3 and 7 days after breast cancer surgery. Decreases in p_*Firmicutes* and f_*Lachnospiraceae* might result in decreased SCFA production. Increases in p_*Proteobacteria* and f_*Enterobacteriaceae* might be associated with disease and antibiotic resistance. Moreover, a significant number of metabolites changed significantly after breast cancer surgery. Therefore, it is necessary to further strengthen multimodal postoperative management, particularly the improvement of the gut microbiota.

## Data availability statement

The data presented in the study are deposited in the SRA (https://www.ncbi.nlm.nih.gov/sra/) repository, accession number PRJNA1110396.

## Ethics statement

The studies involving humans were approved by the study was conducted in accordance with the Helsinki Declaration and approved by the Ethics Committee of the First Affiliated Hospital of Hainan Medical College (2022llzm1175). The studies were conducted in accordance with the local legislation and institutional requirements. The participants provided their written informed consent to participate in this study. The manuscript presents research on animals that do not require ethical approval for their study.

## Author contributions

PF: Funding acquisition, Investigation, Software, Visualization, Writing – original draft, Writing – review & editing. LD: Methodology, Software, Writing – original draft, Writing – review & editing. GD: Methodology, Writing – original draft, Writing – review & editing, Conceptualization, Data curation, Formal analysis, Investigation. CW: Methodology, Writing – original draft, Writing – review & editing, Project administration, Resources, Supervision, Visualization.

## Footnotes

^1^https://cloud.majorbio.com

## Supplementary material

SUPPLEMENTARY FIGURE S1 PLS-DA analysis was performed to analyze the effect of surgery on the β-diversity of gut microbiota of breast cancer patients.

SUPPLEMENTARY FIGURE S2 Metabolite differences between groups were demonstrated by volcano plots.

SUPPLEMENTARY FIGURE S3 ROC analysis to screen characteristic metabolites and their expression. **(A)** Lysylleucine, **(B)** PE(PGD1/18:1(9Z)), **(C)** 20a,20b-dihomo-9-oxo-11R,15S-dihydroxy-5Z,13E-prostadienoic acid, **(D)** N-[(Z)-1,3-dihydroxyoctadec-4-en-2-yl]-6-[(4-nitro-2,1,3-benzoxadiazol-7-yl)amino]hexanamide, **(E)** Jervine, **(F)** (5Z,7E)-(3S)-26,26,26-Trifluoro-27-nor-9,10-seco-5,7,10(19)-cholestatriene-3,25-diol, **(G)** Chapso, **(H)** alpha-terpineol acetate.
