# Supplementary figures and images for: Effect of mastectomy on gut microbiota and its metabolites in patients with breast cancer
Source: Front Microbiol. 2024 May 27;15:1269558. doi: 10.3389/fmicb.2024.1269558 (PMC11163111; doi:10.3389/fmicb.2024.1269558)

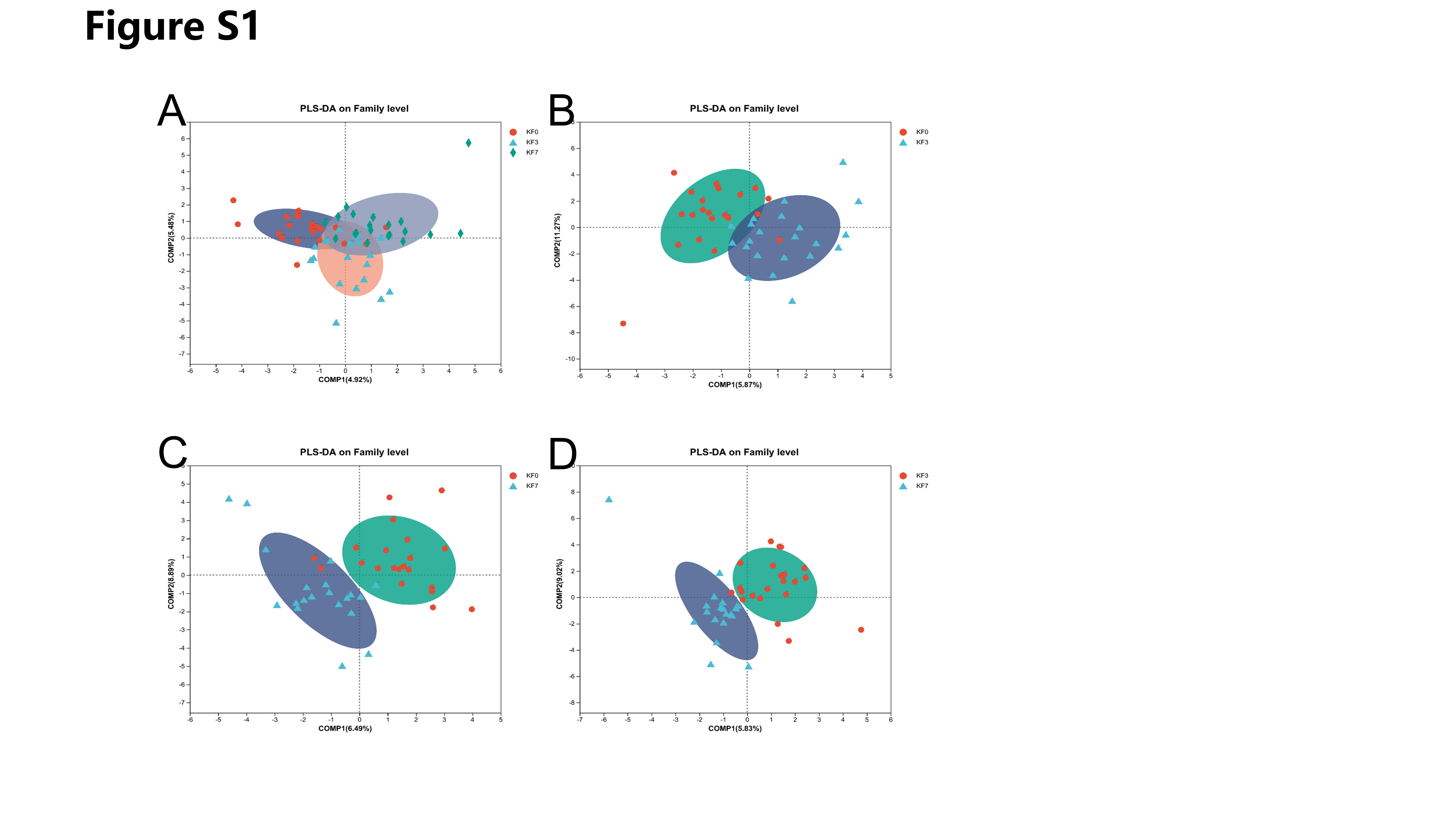

Supplement: SUPPLEMENTARY FIGURE S1 — PLS-DA analysis was performed to analyze the effect of surgery on the β-diversity of gut microbiota of breast cancer patients. [file Image_1.TIF]

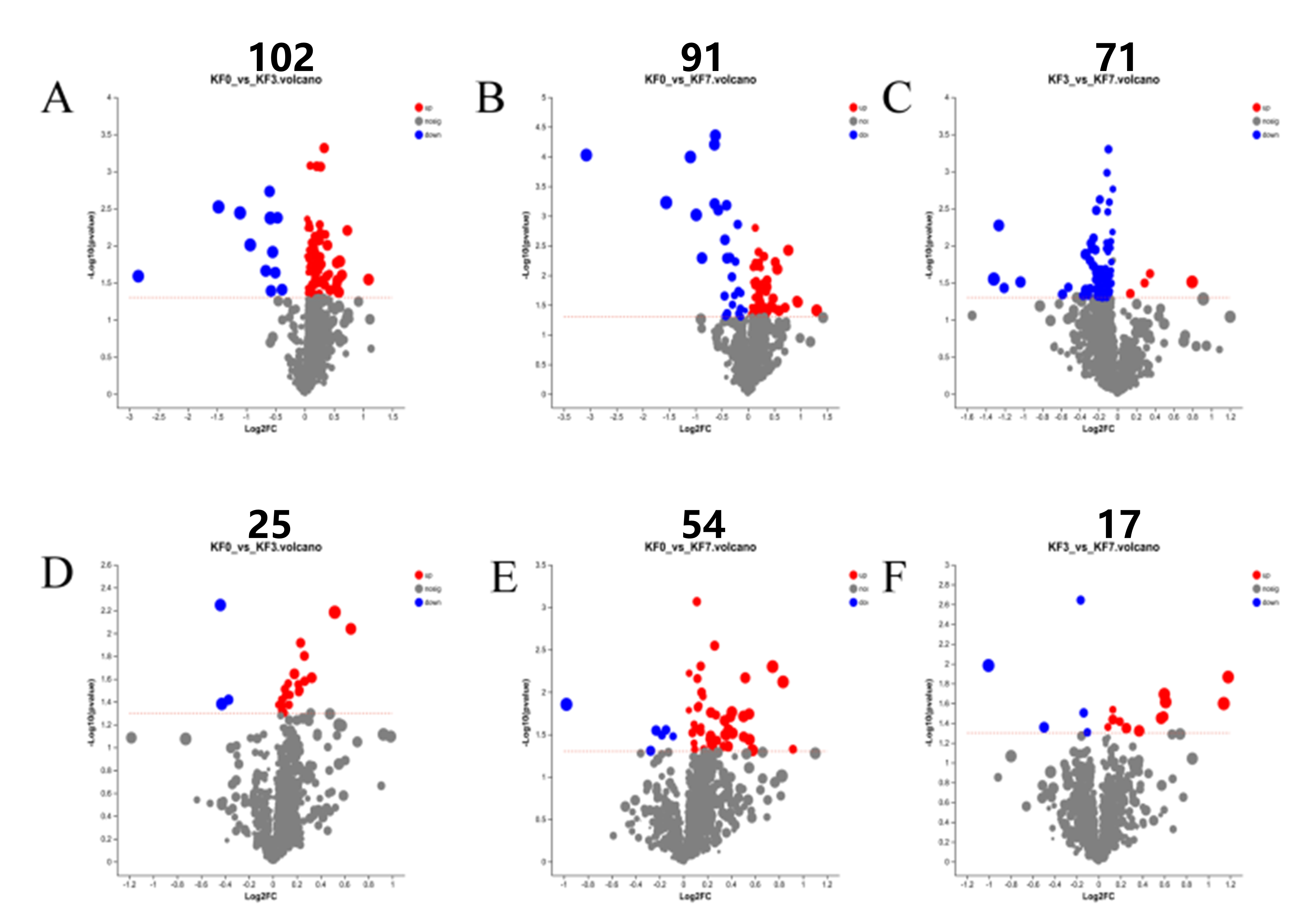

Supplement: SUPPLEMENTARY FIGURE S2 — Metabolite differences between groups were demonstrated by volcano plots. [file Image_2.TIF]

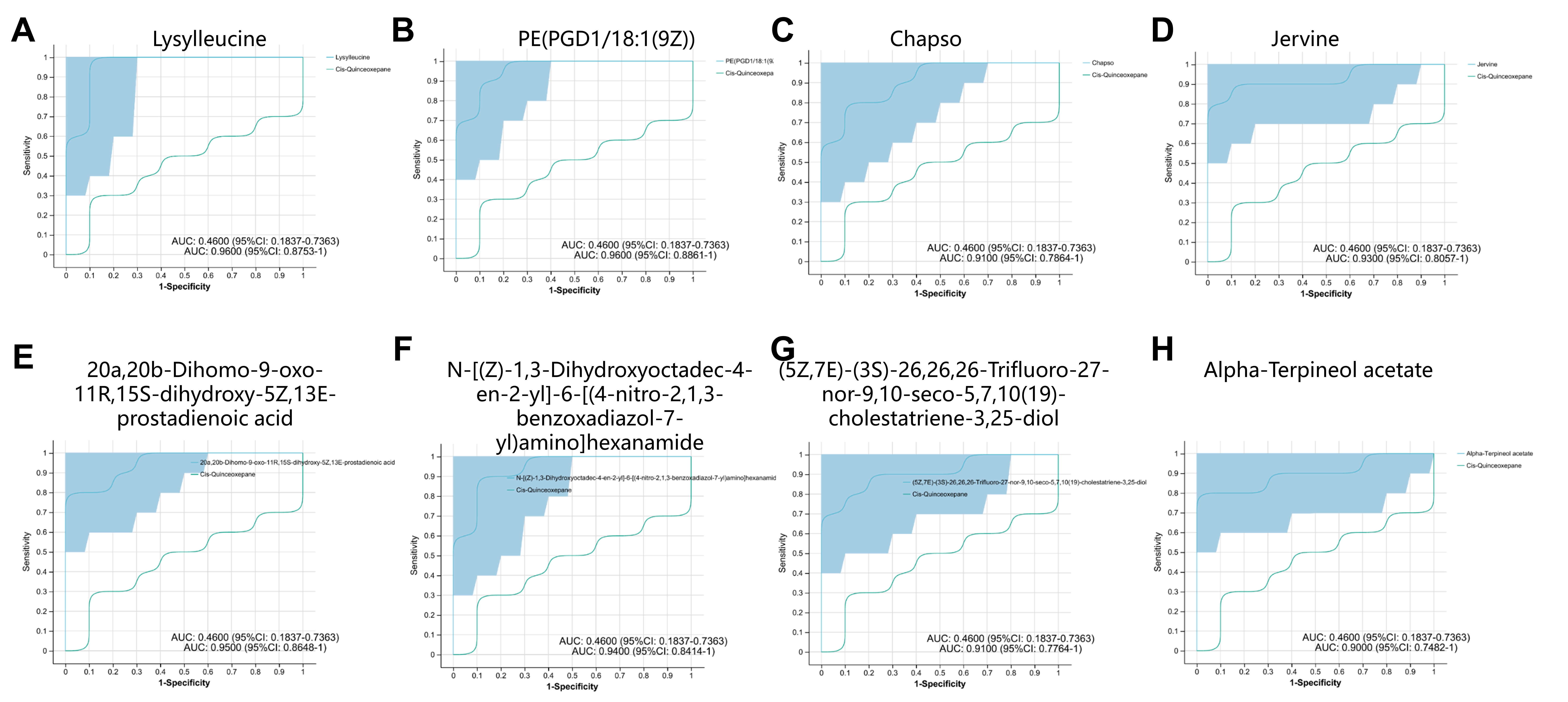

Supplement: SUPPLEMENTARY FIGURE S3 — ROC analysis to screen characteristic metabolites and their expression. (A) Lysylleucine, (B) PE(PGD1/18:1(9Z)), (C) 20a,20b-dihomo-9-oxo-11R,15S-dihydroxy-5Z,13E-prostadienoic acid, (D) N-[(Z)-1,3-dihydroxyoctadec-4-en-2-yl]-6-[(4-nitro-2,1,3-benzoxadiazol-7-yl)amino]hexanamide, (E) Jervine, (F) (5Z,7E)-(3S)-26,26,26-Trifluoro-27-nor-9,10-seco-5,7,10(19)-cholestatriene-3,25-diol, (G) Chapso, (H) alpha-terpineol acetate. [file Image_3.tif]
